# Crystal structure of *catena*-poly[[bis­(acetato-κ*O*)copper(II)]-bis­[μ-4-(1*H*-imidazol-1-yl)phenol]-κ^2^ *N* ^3^:*O*;κ^2^ *O*:*N* ^3^]

**DOI:** 10.1107/S2056989017000780

**Published:** 2017-01-20

**Authors:** Mehmet Poyraz, Musa Sarı

**Affiliations:** a Department of Chemistry, Faculty of Science and Arts, Afyon Kocatepe University, TR-03200 Afyonkarahisar, Turkey; b Department of Physics Education, Gazi University, Beşevler, TR-06500 Ankara, Turkey

**Keywords:** crystal structure, polymeric structure, hydrogen bonds, copper(II), acetate, imidazole, π–π stacking

## Abstract

In the title compound, [Cu(CH_3_COO)_2_(C_9_H_8_N_2_O)_2_]_*n*_, the Cu^II^ ion resides on a centre of inversion, displaying a tetra­gonally distorted octa­hedral coordination environment defined by two pairs of N and O atoms of symmetry-related 4-(1*H*-imidazol-1-yl)phenol ligands and the O atoms of two symmetry-related acetate ligands. The bridging mode of the 4-(1*H*-imidazol-1-yl)phenol ligands is associated with a very long Cu⋯O inter­actions involving the phenol O atom of the heterocyclic ligand, which creates chains extending parallel to [100]. In the crystal, the chains are arranged in a distorted hexa­gonal rod packing and are linked *via* C—H⋯O hydrogen bonds and by π–π stacking inter­actions involving centrosymmetrically related pairs of imidazole and phenol rings.

## Abstract 2

The title compound exhibits a polymeric structure caused by long Cu⋯O inter­actions, which lead to the formation of chains parallel to [100]. In the crystal, the chains are arranged in a distorted hexa­gonal rod packing.

## Chemical context   

Coordination polymers have been investigated as materials with inter­esting properties such as magnetism (Zhu *et al.*, 2010 ▸), luminescence (Cui *et al.*, 2012 ▸), catalysis (Wang *et al.*, 2011 ▸) or absorption (Zhang *et al.*, 2017 ▸). Some coordination polymers are also known to show photocatalytic activity with respect to the decomposition of organic dyes (Yang *et al.*, 2010 ▸; Yin *et al.*, 2015 ▸).
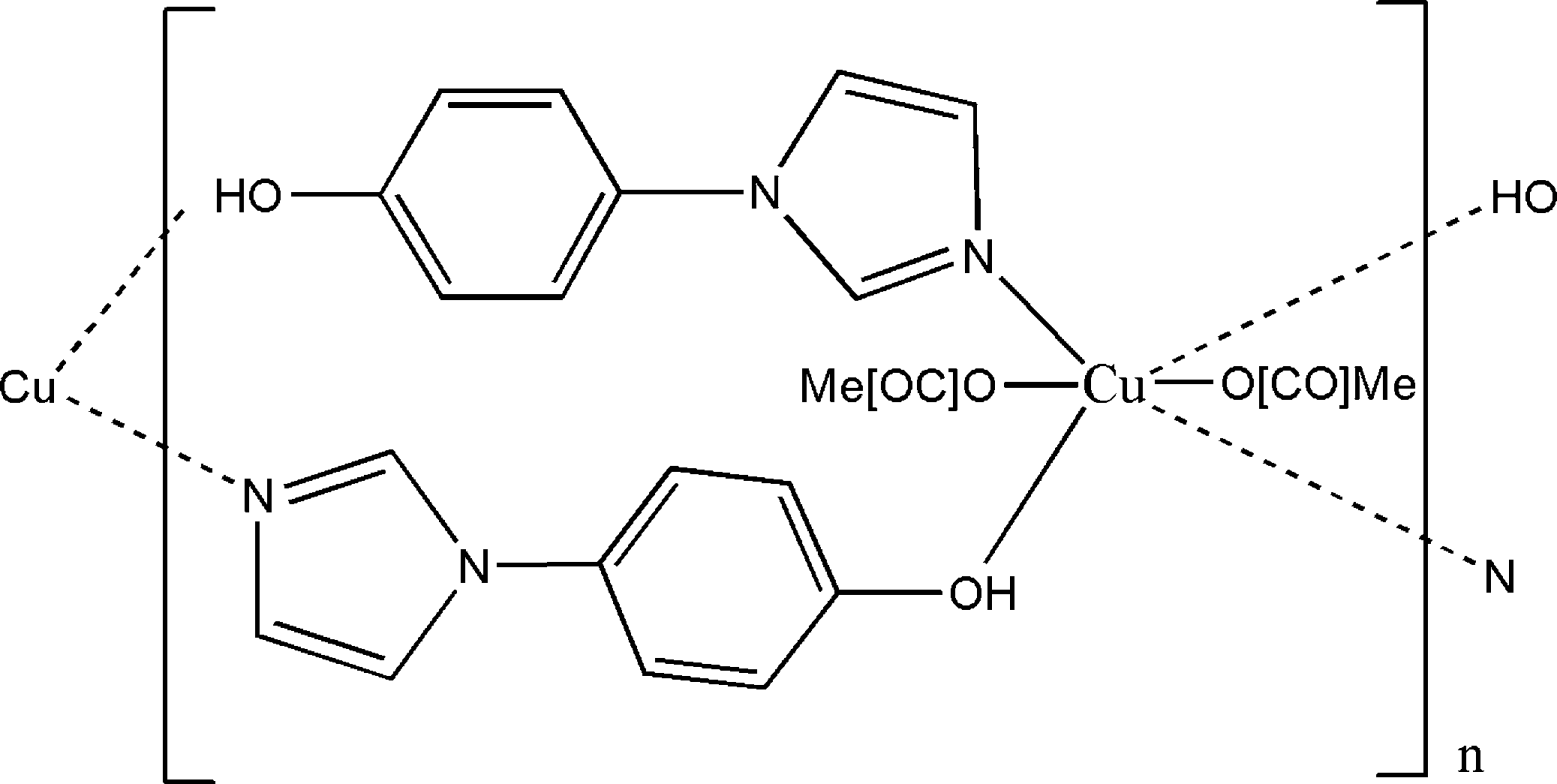


In the past few years, metal complexes with ligands derived from imidazole have attracted much attention, not only for their fascinating crystal structures, but also for their inter­esting applications related to anti­fungal (Rezaei *et al.*, 2011 ▸), pesticidal (Stenersen *et al.*, 2004 ▸) and plant-growth regulatory properties (Choi *et al.*, 2010 ▸), or drugs in general (Lednicer *et al.*, 1998 ▸; Adams *et al.*, 2001 ▸). Most of these compounds exhibit typical mol­ecular structures whereas the number of imidazole-based coordination polymers (Martins *et al.*, 2010 ▸; Masciocchi *et al.*, 2001 ▸; Stamatatos *et al.*, 2009 ▸) is much lower, probably due to the difficulty of growing single crystals.

In this communication we report on the synthesis and crystal structure of a copper(II) coordination polymer, [Cu(CH_3_COO)_2_(C_9_H_8_N_2_O)_2_]_*n*_, comprising 4-(1*H*-imidazol-1-yl)-phenol and acetate ligands.

## Structural commentary   

**Figure 1:**
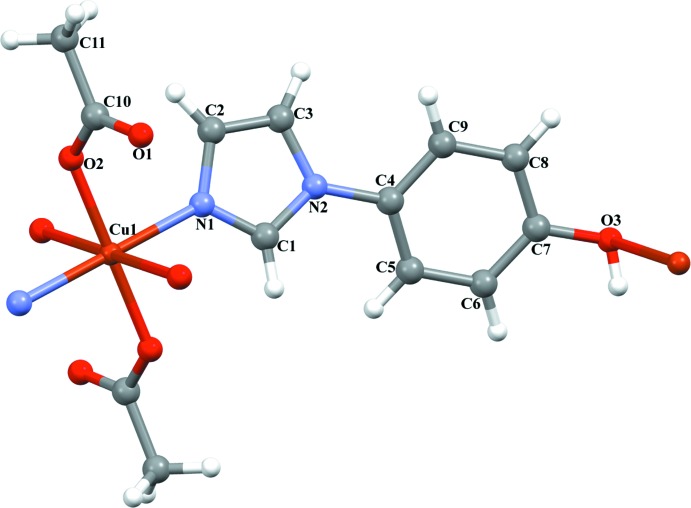
The coordination environment of the Cu^II^ atom in the title compound. Displacement ellipsoids are drawn at the 30% probability level; non-labelled atoms are related to labelled atoms by (−*x* + 1, −*y* + 1, −*z*).

**Figure 2:**
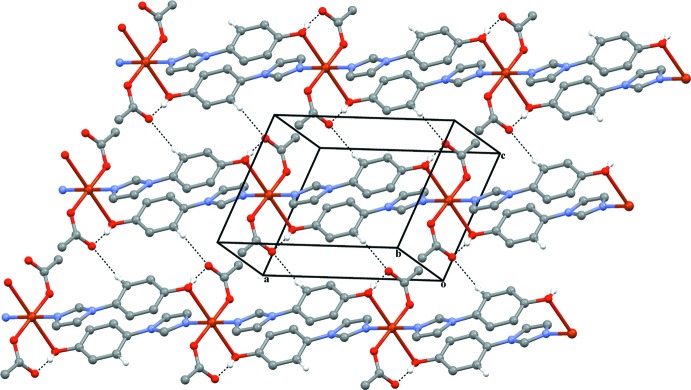
The crystal structure of the title compound showing the formation of chains extending parallel to [100]. Hydrogen-bonding inter­actions are shown as dashed lines.

The asymmetric unit of the title compound comprises of one Cu^II^ atom, one 4-(1*H*-imidazol-1-yl)-phenol ligand and one acetate group, with the Cu^II^ atom situated on a crystallographic inversion centre. The distorted octa­hedral coordination environment of the Cu^II^ atom is defined by two symmetry-related pairs of imidazole N atoms and phenol O atoms from the heterocyclic ligands and by two O atoms of a symmetry-related pair of monodentate acetate ligands (Fig. 1 ▸). The Cu—O(acetate) [1.9322 (18) Å] and Cu—N(imidazole) [2.003 (2) Å] bonds are arranged in the equatorial plane and are within normal lengths (Ding *et al.*, 2005 ▸; Song *et al.*, 2008 ▸; Yun *et al.*, 2008 ▸; Yu & Deng, 2011 ▸). The equatorial bond angles are in the range 86.94 (7)–93.06 (7)° in the Cu1N_2_O_4_ polyhedron (Table 1 ▸). The bond involving the phenolic O3 atom is very weak, with a distance of Cu⋯O = 2.739 (2) Å, completing the tetra­gonally distorted octa­hedron. The *N*,*O*-bridging character of the 4-(1*H*-imidazol-1-yl)-phenol ligand leads to the formation of chains extending parallel to [100], whereby the ligands are oriented in an anti­parallel fashion within a chain. The dihedral angle between the imidazole group (N1,N2,C1–C3) and the phenyl ring (C4–C9) is 24.07 (2)°. An intra­chain hydrogen bond between the phenol OH group (O3) and the non-coordinating carboxyl­ate O atom (O1) of the acetate ligand is present (Table 2 ▸, Fig. 2 ▸).

## Supra­molecular features   

In the crystal, the chains are aligned in a distorted hexa­gonal rod packing perpendicular to the chain direction. Chains are linked through inter­molecular C—H⋯O inter­actions between a phenyl CH group and the non-coordinating carboxyl­ate O atom (O1) that consequently acts as a double acceptor atom (Fig. 2 ▸, Table 2 ▸). Additional π–π stacking inter­actions involving centrosymmetrically related pairs of imidazole and phenol rings, with the shortest distance between an N atom and a C atom being 3.372 (2) Å, are also present. The inter­planar angle between the two rings is 24.1 (1)°.

## Database survey   

The literature about one-dimensional inorganic–organic coordination polymers based on copper(II) complexes with Cu^II^ either in a square-pyramidal or a distorted octa­hedral coordination environment is vast. Just to take very recent examples, three such structures have been reported (Hazra *et al.*, 2017 ▸; Puchoňová *et al.*, 2017 ▸; Shaabani *et al.*, 2017 ▸). Nevertheless, there is only limited research on 4-(1*H*-imidazol-1-yl)-phenol as a ligand (Maher *et al.*, 1994 ▸; Wei *et al.*, 2007 ▸; Yurdakul & Badoğlu, 2015 ▸). To the best of our know­ledge, only one discrete copper(II) complex of 4-(1*H*-imidazol-1-yl)-phenol (Yu & Deng, 2011 ▸) has been reported. In this regard, the title compound is the first Cu^II^ coordination polymer with 4-(1*H*-imidazol-1-yl)-phenol.

## Synthesis and crystallization   

4-(1*H*-Imidazol-1-yl)phenol (0.0480 g, 0.3 mmol) was dissolved in 5 ml ethanol, a water solution (5 ml) of Na_2_CO_3_ (0.0318 g, 0.3 mmol) was slowly added, and an ethanol solution (5 ml) of Cu(NO_3_)_2_·2.5H_2_O (0.0349 g, 0.15 mmol) was added slowly with stirring for 30 min. To the formed cloudy suspension, an aqueous solution of acetic acid (0.3 mmol) was added. The resulting solution turned to a transparent blue colour. After stirring for three h, the solution was allowed to evaporate at room temperature. A number of blue single crystals were obtained after a few days.

## Refinement   

Crystal data, data collection and structure refinement details are summarized in Table 3 ▸. C-bound H atoms were placed in geometrically idealized positions and constrained to ride on their parent atoms with distances in the range 0.93–0.96Å and with *U*_iso_(H) = 1.2*U*_eq_(C) or 1.5*U*_eq_(C) for methyl atoms. The H atom of the phenol OH group was located in a difference map and was constrained at a distance of O—H = 0.84 Å and with *U*_iso_(H) =1.5*U*_eq_(O).

## Supplementary Material

Crystal structure: contains datablock(s) I, Global. DOI: 10.1107/S2056989017000780/wm4035sup1.cif

Structure factors: contains datablock(s) I. DOI: 10.1107/S2056989017000780/wm4035Isup2.hkl

CCDC reference: 1520352

Additional supporting information:  crystallographic information; 3D view; checkCIF report

## Figures and Tables

**Table 1:** Selected geometric parameters (Å, °)

N1—Cu1	2.003 (2)	O3—Cu1^i^	2.739 (2)
Cu1—O2	1.9322 (18)		
			
O2—Cu1—N1	90.56 (8)	N1—Cu1—O3^iii^	91.31 (7)
N1—Cu1—N1^ii^	180.0	O2—Cu1—O3^iv^	86.94 (7)
O2—Cu1—O3^iii^	93.06 (7)	N1—Cu1—O3^iv^	88.69 (7)

Symmetry codes: (i) 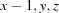; (ii) 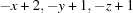; (iii) 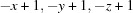; (iv) 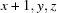.

**Table 2:** Hydrogen-bond geometry (Å, °)

*D*—H⋯*A*	*D*—H	H⋯*A*	*D*⋯*A*	*D*—H⋯*A*
C5—H5⋯O1^v^	0.95	2.44	3.356 (3)	161
O3—H3*A*⋯O1^iii^	0.84	1.80	2.637 (3)	172

Symmetry codes: (iii) 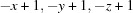; (v) 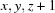.

**Table 3:** Experimental details

Crystal data	
Chemical formula	[Cu(C_2_H_3_O_2_)_2_(C_9_H_8_N_2_O)_2_]
*M*_r_	501.99
Crystal system, space group	Monoclinic, *P*2_1_/*c*
Temperature (K)	296
*a*, *b*, *c* (Å)	10.2029 (15), 15.089 (2), 7.7814 (11)
β (°)	111.545 (4)
*V* (Å^3^)	1114.2 (3)
*Z*	2
Radiation type	Mo *K*α
μ (mm^−1^)	1.03
Crystal size (mm)	0.11 × 0.09 × 0.07

Data collection	
Diffractometer	Bruker APEXII CCD
Absorption correction	Multi-scan (*SADABS*; Bruker, 2013 ▸)
*T*_min_, *T*_max_	0.895, 0.931
No. of measured, independent and observed [*I* > 2σ(*I*)] reflections	40729, 2784, 2156
*R*_int_	0.051
(sin θ/λ)_max_ (Å^−1^)	0.667

Refinement	
*R*[*F* ^2^ > 2σ(*F* ^2^)], *wR*(*F* ^2^), *S*	0.041, 0.100, 1.15
No. of reflections	2784
No. of parameters	153
H-atom treatment	H-atom parameters constrained
Δρ_max_, Δρ_min_ (e Å^−3^)	0.26, −0.30

Computer programs: *APEX2* and *SAINT* (Bruker, 2013 ▸), *SHELXS97* (Sheldrick, 2008 ▸), *SHELXL2014* (Sheldrick, 2015 ▸), *Mercury* (Macrae *et al.*, 2008 ▸) and *publCIF* (Westrip, 2010 ▸).

## supplementary crystallographic information

### Crystal data

**Table:**

[Cu(C_2_H_3_O_2_)_2_(C_9_H_8_N_2_O)_2_]	*F*(000) = 518
*M**_r_* = 501.99	*D*_x_ = 1.496 Mg m^−^^3^
Monoclinic, *P*2_1_/*c*	Mo *K*α radiation, λ = 0.71073 Å
*a* = 10.2029 (15) Å	Cell parameters from 9911 reflections
*b* = 15.089 (2) Å	θ = 3.2–28.0°
*c* = 7.7814 (11) Å	µ = 1.03 mm^−^^1^
β = 111.545 (4)°	*T* = 296 K
*V* = 1114.2 (3) Å^3^	Block, blue
*Z* = 2	0.11 × 0.09 × 0.07 mm

### Data collection

**Table:**

Bruker APEXII CCD diffractometer	2156 reflections with *I* > 2σ(*I*)
φ and ω scans	*R*_int_ = 0.051
Absorption correction: multi-scan (*SADABS*; Bruker, 2013)	θ_max_ = 28.3°, θ_min_ = 3.1°
*T*_min_ = 0.895, *T*_max_ = 0.931	*h* = −13→13
40729 measured reflections	*k* = −20→20
2784 independent reflections	*l* = −10→10

### Refinement

**Table:**

Refinement on *F*^2^	0 restraints
Least-squares matrix: full	Hydrogen site location: inferred from neighbouring sites
*R*[*F*^2^ > 2σ(*F*^2^)] = 0.041	H-atom parameters constrained
*wR*(*F*^2^) = 0.100	*w* = 1/[σ^2^(*F*_o_^2^) + (0.0249*P*)^2^ + 1.2448*P*] where *P* = (*F*_o_^2^ + 2*F*_c_^2^)/3
*S* = 1.15	(Δ/σ)_max_ < 0.001
2784 reflections	Δρ_max_ = 0.26 e Å^−^^3^
153 parameters	Δρ_min_ = −0.30 e Å^−^^3^

### Special details

**Table:**

Geometry. All esds (except the esd in the dihedral angle between two l.s. planes) are estimated using the full covariance matrix. The cell esds are taken into account individually in the estimation of esds in distances, angles and torsion angles; correlations between esds in cell parameters are only used when they are defined by crystal symmetry. An approximate (isotropic) treatment of cell esds is used for estimating esds involving l.s. planes.

### Fractional atomic coordinates and isotropic or equivalent isotropic displacement parameters (Å^2^)

**Table:**

	*x*	*y*	*z*	*U*_iso_*/*U*_eq_	
C1	0.7431 (2)	0.55700 (18)	0.5692 (3)	0.0350 (5)	
H1	0.7461	0.5059	0.6420	0.042*	
C2	0.7933 (4)	0.6526 (2)	0.4009 (5)	0.0601 (9)	
H2	0.8394	0.6819	0.3308	0.072*	
C3	0.6778 (4)	0.6824 (2)	0.4266 (5)	0.0656 (10)	
H3	0.6285	0.7359	0.3794	0.079*	
C4	0.5265 (2)	0.62360 (16)	0.5918 (3)	0.0333 (5)	
C5	0.5302 (3)	0.57796 (19)	0.7472 (4)	0.0408 (6)	
H5	0.6125	0.5463	0.8195	0.049*	
C6	0.4129 (3)	0.5787 (2)	0.7968 (4)	0.0430 (6)	
H6	0.4149	0.5470	0.9033	0.052*	
C7	0.2929 (2)	0.62494 (18)	0.6930 (3)	0.0370 (6)	
C8	0.2926 (3)	0.67390 (18)	0.5434 (4)	0.0419 (6)	
H8	0.2126	0.7087	0.4760	0.050*	
C9	0.4084 (3)	0.67258 (18)	0.4912 (4)	0.0403 (6)	
H9	0.4069	0.7053	0.3861	0.048*	
C10	0.9356 (3)	0.56669 (19)	0.1300 (3)	0.0402 (6)	
C11	0.9592 (4)	0.6405 (2)	0.0154 (4)	0.0617 (9)	
H11A	0.9094	0.6273	−0.1159	0.093*	
H11B	1.0603	0.6463	0.0408	0.093*	
H11C	0.9234	0.6961	0.0464	0.093*	
N1	0.8345 (2)	0.57396 (15)	0.4905 (3)	0.0368 (5)	
N2	0.6450 (2)	0.62096 (14)	0.5338 (3)	0.0362 (5)	
Cu1	1.0000	0.5000	0.5000	0.03750 (14)	
O1	0.8401 (2)	0.51168 (15)	0.0597 (3)	0.0528 (5)	
O2	1.01849 (18)	0.56716 (14)	0.2987 (2)	0.0455 (5)	
O3	0.17326 (19)	0.62330 (15)	0.7320 (3)	0.0502 (5)	
H3A	0.1775	0.5811	0.8043	0.075*	

### Atomic displacement parameters (Å^2^)

**Table:**

	*U*^11^	*U*^22^	*U*^33^	*U*^12^	*U*^13^	*U*^23^
C1	0.0274 (11)	0.0431 (14)	0.0347 (12)	−0.0002 (10)	0.0115 (9)	0.0037 (10)
C2	0.073 (2)	0.0432 (17)	0.089 (2)	0.0039 (15)	0.060 (2)	0.0134 (16)
C3	0.079 (2)	0.0395 (17)	0.104 (3)	0.0157 (16)	0.064 (2)	0.0252 (17)
C4	0.0288 (11)	0.0337 (12)	0.0384 (13)	−0.0008 (9)	0.0135 (10)	−0.0017 (10)
C5	0.0271 (12)	0.0540 (17)	0.0403 (13)	0.0071 (11)	0.0111 (10)	0.0107 (12)
C6	0.0345 (13)	0.0601 (18)	0.0369 (13)	0.0048 (12)	0.0161 (11)	0.0117 (12)
C7	0.0282 (11)	0.0441 (14)	0.0403 (13)	0.0003 (10)	0.0144 (10)	−0.0056 (11)
C8	0.0333 (13)	0.0411 (15)	0.0494 (15)	0.0096 (11)	0.0129 (11)	0.0067 (12)
C9	0.0380 (13)	0.0397 (14)	0.0440 (14)	0.0047 (11)	0.0159 (11)	0.0092 (11)
C10	0.0364 (13)	0.0524 (16)	0.0369 (13)	0.0142 (12)	0.0195 (11)	0.0071 (12)
C11	0.082 (2)	0.056 (2)	0.0539 (18)	0.0127 (17)	0.0328 (17)	0.0158 (15)
N1	0.0313 (10)	0.0428 (12)	0.0393 (11)	−0.0035 (9)	0.0165 (9)	−0.0029 (9)
N2	0.0333 (10)	0.0357 (11)	0.0435 (11)	0.0015 (9)	0.0187 (9)	0.0027 (9)
Cu1	0.0246 (2)	0.0589 (3)	0.0293 (2)	0.0002 (2)	0.01022 (15)	0.0018 (2)
O1	0.0430 (10)	0.0701 (14)	0.0416 (10)	0.0012 (10)	0.0112 (8)	0.0052 (10)
O2	0.0316 (9)	0.0717 (14)	0.0350 (9)	0.0000 (9)	0.0141 (8)	0.0087 (9)
O3	0.0339 (9)	0.0667 (14)	0.0571 (12)	0.0073 (9)	0.0253 (9)	0.0065 (10)

### Geometric parameters (Å, º)

**Table:**

C1—N1	1.315 (3)	C8—C9	1.383 (4)
C1—N2	1.345 (3)	C8—Cu1^i^	3.895 (3)
C1—Cu1	2.989 (2)	C8—H8	0.9500
C1—H1	0.9500	C9—H9	0.9500
C2—C3	1.343 (4)	C10—O1	1.244 (3)
C2—N1	1.361 (4)	C10—O2	1.274 (3)
C2—Cu1	3.024 (3)	C10—C11	1.501 (4)
C2—H2	0.9500	C10—Cu1	2.888 (3)
C3—N2	1.369 (4)	C11—H11A	0.9800
C3—H3	0.9500	C11—H11B	0.9800
C4—C5	1.380 (3)	C11—H11C	0.9800
C4—C9	1.385 (3)	N1—Cu1	2.003 (2)
C4—N2	1.438 (3)	Cu1—O2^ii^	1.9322 (18)
C5—C6	1.386 (3)	Cu1—O2	1.9322 (18)
C5—H5	0.9500	Cu1—N1^ii^	2.003 (2)
C6—C7	1.383 (4)	Cu1—O3^iii^	2.739 (2)
C6—H6	0.9500	Cu1—O3^iv^	2.739 (2)
C7—O3	1.363 (3)	O3—Cu1^i^	2.739 (2)
C7—C8	1.377 (4)	O3—H3A	0.8400
C7—Cu1^i^	3.383 (2)		
			
N1—C1—N2	111.5 (2)	O1—C10—O2	125.0 (3)
N2—C1—Cu1	143.71 (17)	O1—C10—C11	120.3 (3)
N1—C1—H1	124.2	O2—C10—C11	114.7 (3)
N2—C1—H1	124.2	O1—C10—Cu1	93.50 (16)
Cu1—C1—H1	92.1	C11—C10—Cu1	145.5 (2)
C3—C2—N1	109.8 (3)	C10—C11—H11A	109.5
C3—C2—Cu1	141.7 (2)	C10—C11—H11B	109.5
C3—C2—H2	125.1	H11A—C11—H11B	109.5
N1—C2—H2	125.1	C10—C11—H11C	109.5
Cu1—C2—H2	93.2	H11A—C11—H11C	109.5
C2—C3—N2	106.8 (3)	H11B—C11—H11C	109.5
C2—C3—H3	126.6	C1—N1—C2	105.7 (2)
N2—C3—H3	126.6	C1—N1—Cu1	127.34 (18)
C5—C4—C9	120.0 (2)	C2—N1—Cu1	127.00 (18)
C5—C4—N2	120.4 (2)	C1—N2—C3	106.3 (2)
C9—C4—N2	119.6 (2)	C1—N2—C4	127.2 (2)
C4—C5—C6	119.4 (2)	C3—N2—C4	126.5 (2)
C4—C5—H5	120.3	O2^ii^—Cu1—O2	180.0
C6—C5—H5	120.3	O2^ii^—Cu1—N1	89.44 (8)
C7—C6—C5	120.7 (2)	O2—Cu1—N1	90.56 (8)
C7—C6—H6	119.6	O2^ii^—Cu1—N1^ii^	90.56 (8)
C5—C6—H6	119.6	O2—Cu1—N1^ii^	89.44 (8)
O3—C7—C8	118.3 (2)	N1—Cu1—N1^ii^	180.0
O3—C7—C6	122.3 (2)	O2^ii^—Cu1—O3^iii^	86.94 (7)
C8—C7—C6	119.4 (2)	O2—Cu1—O3^iii^	93.06 (7)
O3—C7—Cu1^i^	51.02 (13)	N1—Cu1—O3^iii^	91.31 (7)
C8—C7—Cu1^i^	101.33 (16)	N1^ii^—Cu1—O3^iii^	88.69 (7)
C6—C7—Cu1^i^	115.23 (18)	O2^ii^—Cu1—O3^iv^	93.06 (7)
C7—C8—C9	120.2 (2)	O2—Cu1—O3^iv^	86.94 (7)
C7—C8—Cu1^i^	58.39 (14)	N1—Cu1—O3^iv^	88.69 (7)
C9—C8—Cu1^i^	132.43 (19)	N1^ii^—Cu1—O3^iv^	91.31 (7)
C7—C8—H8	119.9	O3^iii^—Cu1—O3^iv^	180.00 (7)
C9—C8—H8	119.9	C10—O1—Cu1	63.78 (14)
Cu1^i^—C8—H8	81.3	C10—O2—Cu1	127.36 (18)
C8—C9—C4	120.1 (2)	C7—O3—Cu1^i^	106.23 (16)
C8—C9—H9	120.0	C7—O3—H3A	109.5
C4—C9—H9	120.0	Cu1^i^—O3—H3A	77.8
			
N1—C2—C3—N2	−0.4 (4)	C3—C2—N1—C1	0.1 (4)
Cu1—C2—C3—N2	−0.5 (6)	Cu1—C2—N1—C1	179.9 (3)
C9—C4—C5—C6	2.6 (4)	C3—C2—N1—Cu1	−179.8 (2)
N2—C4—C5—C6	−177.6 (3)	N1—C1—N2—C3	−0.4 (3)
C4—C5—C6—C7	−0.3 (4)	Cu1—C1—N2—C3	−0.5 (4)
C5—C6—C7—O3	176.5 (3)	N1—C1—N2—C4	177.9 (2)
C5—C6—C7—C8	−2.9 (4)	Cu1—C1—N2—C4	177.83 (19)
C5—C6—C7—Cu1^i^	118.1 (3)	C2—C3—N2—C1	0.5 (4)
O3—C7—C8—C9	−175.6 (3)	C2—C3—N2—C4	−177.9 (3)
C6—C7—C8—C9	3.8 (4)	C5—C4—N2—C1	25.2 (4)
Cu1^i^—C7—C8—C9	−123.9 (2)	C9—C4—N2—C1	−155.1 (3)
O3—C7—C8—Cu1^i^	−51.63 (19)	C5—C4—N2—C3	−156.8 (3)
C6—C7—C8—Cu1^i^	127.7 (3)	C9—C4—N2—C3	23.0 (4)
C7—C8—C9—C4	−1.5 (4)	O2—C10—O1—Cu1	6.7 (2)
Cu1^i^—C8—C9—C4	−74.7 (3)	C11—C10—O1—Cu1	−172.5 (3)
C5—C4—C9—C8	−1.7 (4)	O1—C10—O2—Cu1	−12.6 (4)
N2—C4—C9—C8	178.5 (2)	C11—C10—O2—Cu1	166.60 (19)
N2—C1—N1—C2	0.2 (3)	C8—C7—O3—Cu1^i^	81.4 (3)
Cu1—C1—N1—C2	−179.9 (3)	C6—C7—O3—Cu1^i^	−97.9 (3)
N2—C1—N1—Cu1	−179.88 (16)		

Symmetry codes: (i) *x*−1, *y*, *z*; (ii) −*x*+2, −*y*+1, −*z*+1; (iii) −*x*+1, −*y*+1, −*z*+1; (iv) *x*+1, *y*, *z*.

### Hydrogen-bond geometry (Å, º)

**Table:**

*D*—H···*A*	*D*—H	H···*A*	*D*···*A*	*D*—H···*A*
C5—H5···O1^v^	0.95	2.44	3.356 (3)	161
O3—H3*A*···O1^iii^	0.84	1.80	2.637 (3)	172

Symmetry codes: (iii) −*x*+1, −*y*+1, −*z*+1; (v) *x*, *y*, *z*+1.
